# Not Just a Medical Student: Delivering Medical Education Through a Short Video Series on Social Media

**DOI:** 10.2196/11971

**Published:** 2019-05-06

**Authors:** Nadine Abbas, Utkarsh Ojha

**Affiliations:** 1 Faculty of Medicine University of Southampton Southampton United Kingdom; 2 Faculty of Medicine Imperial College London London United Kingdom

**Keywords:** social media, medical student, medical education, innovation, videos, Facebook

## Abstract

“Not Just a Medical Student” is an innovative bite-size medical education video series founded and hosted on social media. Its primary aim is to inspire tomorrow’s doctors to be creative while engaging and informing them with the latest innovations, technology, and conferences within various specialties. To our knowledge, these themes are scarcely covered in the structured medical curriculum. Created and launched in August 2017, “Not Just a Medical Student” quickly gained traction; with over 1000 followers on Facebook and a rapidly increasing number of views, it reached the medical community across the globe. The video series features a trailblazer in virtual reality surgery and its potential impact on the evolution of medical education, reviewing future medical technology apps, such as Touch Surgery, and reporting on the latest medical education and health apps. The series engaged in topical medico-politics at the British Medical Association House and reported on global health issues and innovations at the Royal Society of Medicine Conference. The video series has further received several national awards including the *Association and Study of Medical Education (ASME) Educator Innovator 2017* award, runner up to the *Zeshan Qureshi Outstanding Contribution to Medical Education Award*, and the *Alternative Docs National Social Media Influencer* award. The concept has been presented at international conferences (eg, the Healthcare Leadership Academy conference) and gained international recognition upon personal invitation at the Norwegian Annual Junior Doctors Conference. With the rise of the social media generation, innovative methods to inspire, engage, and inform students contributing to the continuous evolution of medical education should be encouraged and further explored.

## Introduction

From our experience, themes on medical innovation and entrepreneurship are rarely covered in the current saturated medical curriculum. To inspire tomorrow’s doctors to be creative and entrepreneurial, there is a need to engage them with the current leaders in the medical profession and the latest technological innovations. For example, the relevance and benefits of leadership and management skills for doctors to enhance patient outcomes, have, in recent years, become more apparent [1]. Many reports, frameworks, and organizations have been created to increase efforts to evolve medical education to reflect such need [2,3]. However, changing the currently saturated medical curriculum to accommodate additions remains challenging [4].

With the rise of the “social media generation,” social media platforms have become an integral part of communication for medical students across the world. Online platforms provide an innovative avenue to educate medical students about current developments and research within the medical profession. There has been a growing trend on the use of social media to aid education, be it through Twitter, Instagram, Facebook, or LinkedIn [5-7]. The integration of medical education into social media can enhance the student experience by taking advantage of a platform to aid educators to connect, debate, inform on topics, and distribute educational materials in an instant. Without limitations of location or time, virtual communities are formed. Social media is an equal playing field for any student to contribute to with ease of accessibility.

The learning experience of videos for medical education on platforms such as YouTube in the literature has previously been published [8,9]. A study on 1083 Australian medical students showed that the vast majority (92%) use online teaching videos to supplement their learning [10]. The use of videos is now becoming a mainstay of medical education. Therefore, we questioned how we can use videos and social media to bridge the gap with topics currently not covered in the core medical curriculum.

## Prior Work

**Figure 1:**
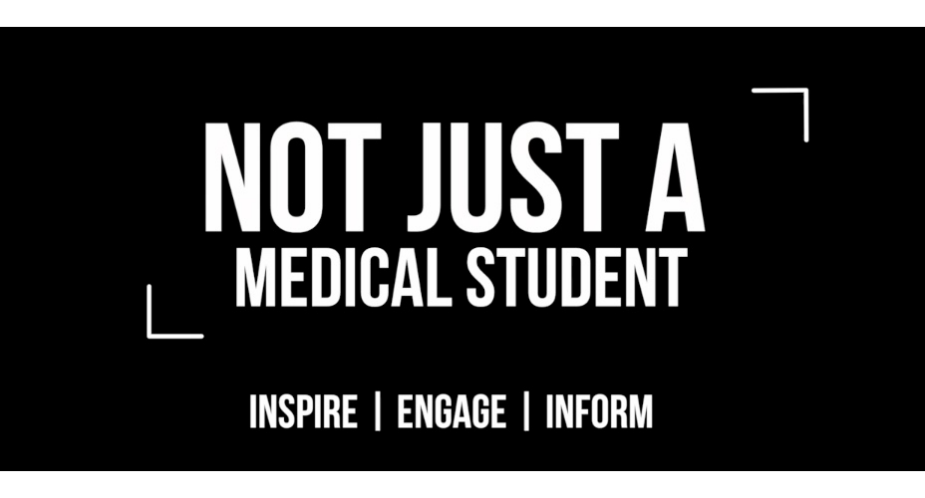
"Not Just a Medical Student" design.

“Not Just a Medical Student” is a multi-award winning, innovative, bite-size medical education video series that was created and launched by a medical student in August 2017 on Facebook (Figure 1) [11]. It seeks to inspire, engage, and inform tomorrow’s doctors with high-quality videos about the latest developments in medicine and surgery in order to combat the lack of exposure found in the core medical curriculum. It aimed to use an established platform to be able to enhance the learning experience and collaboration for students. On inception, it gained traction quickly with over 1000 followers on Facebook, reaching medical students worldwide. To our knowledge, such a concept was never tried before.

“Not Just a Medical Student” aims to replicate short-length videos generally found on social media. These videos were recorded in several different locations including the British Medical Association offices and the Royal Society of Medicine. The video series featured esteemed physicians, surgeons, entrepreneurs including Professor Shafi Ahmed, a trailblazer in virtual reality surgery, and Touch Surgery [12], which is an app that allows a user to practice surgical procedures. The videos discussed and debated a range of topical subjects from global health issues to gender equality in medicine. It finally also leaves the viewers with an engaging take-away message for students. 

The process of creating these videos consisted of gaining prior consent from the establishment or individual featured to film the videos. Once filmed, the videos were edited by an experienced video editor. Scripts were written to aid the video narrations. The final video was then sent for approval before it was published online.

These video series have also featured medical students from universities across the United Kingdom, thus building a strong nationwide network of students interested in medical education and innovation.

## Videos

**Figure 2:**
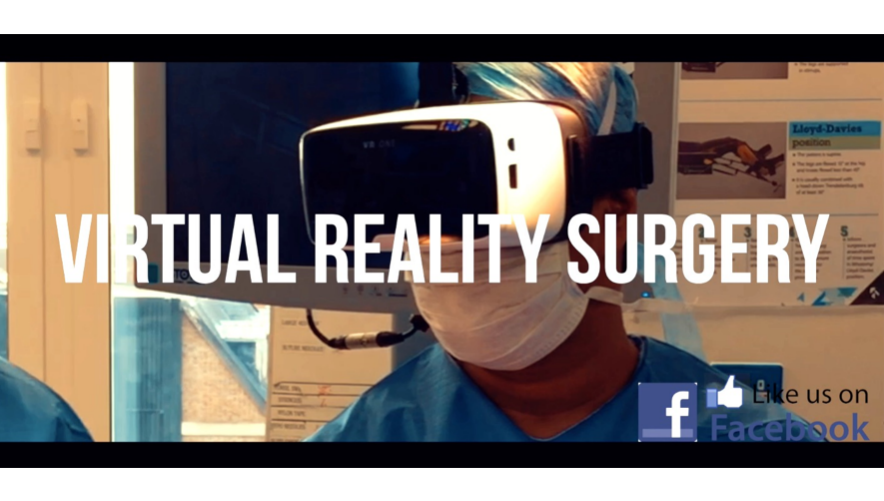
Snapshot of Professor Shafi Ahmed's virtual reality surgery video.

Below, we present examples of the videos released on Facebook:

- “How to learn surgery on a train” - Exploring learning surgery from a mobile app (4700 views)
- “An end to boring hospital placements?” - Exploring innovations by the current generation of students and junior doctors from King’s College London (5100 views)
- “Why is this still happening in the 21st Century?” - Reporting on maternal deaths across the globe and the latest technology set to make a difference (2400 views)
- A personal account and tips on how to publish, from a medical student who managed to obtain 16 publications throughout medical school (2700 views)
- “Will Virtual Reality forever change medical education?”- (3000 views; Figure 2)
- “An increase in medical school places: A detriment to current students?” - Informing and discussing the latest medico-politics currently affecting students (8500 views)
- “From Medical Student to President of The Royal College of Psychiatrists” - An interview with The President, Wendy Burn, on her journey as a medical student, with her take on leadership, resilience, and the future of medical education (3500 views).

## Survey Feedback

We constantly receive comments on how these videos have inspired students to undertake their own projects, and therefore, we created an online survey to anonymously collect responses from viewers as feedback. Respondents of our online feedback survey consisted of medical students as well as nurses, nursing student, doctors, and medical education academics. Our survey showed that all 27 respondents felt that the “Not Just a Medical Student” videos achieved their aims to inspire, engage, and inform medical students. Of the 27 respondents, 24 felt that within the core medical curriculum, there was a gap in the teaching of topics such as medical technology, leadership, and global health. All respondents believed that videos were an effective tool for teaching, and the vast majority (26/27) agreed that social media could be an effective teaching tool. One respondent disagreed and stated that social media was not “sufficiently controlled environment for teaching.” Of the themes explored on “Not Just a Medical Student,” leadership-themed videos were ranked the most beneficial. The vast majority of respondents (26/27) agreed that the topics covered in the videos were relevant to their interests. Our survey and online engagement highlighted an obvious need and appetite for inspiration amongst students via innovative methods found outside of the medical curriculum.

## Recognition

The concept of “Not Just a Medical Student” has won the prestigious *Association and Study of Medical Education (ASME) Innovator Award 2017* [13] and the *Social Media Influencer Award* at the Alternative Docs Conference 2017 for the video series. Additionally, it won runner up for the *Zeshan Qureshi Outstanding Contribution to Medical Education Award*.

International recognition was achieved through personal invitation to present and host a workshop on “Not Just a Medical Student” at the Norwegian Annual Junior Doctors Conference—Yngre legers forening. The video series was orally presented at the ASME Annual Conference 2018 and received high praise. It was also accepted for oral presentation at the Healthcare Leadership Academy Conference 2017 [3].

## Lessons Learned

Creating an ongoing video series on a social media platform has been a new and exciting experience. We realized that for videos, many factors contribute to gaining traction on a platform, such as the number of likes, clicks, shares, and comments. Therefore, the initial period required persistent effort to have our colleagues and peers spread the videos.

Releasing videos on a regular basis and maintaining the high-impact quality of videos requires multiple skilled videographers. Therefore, balancing a medical degree with videography required effective time-management skills.

Despite the advantages of social media previously expressed, the current absence of universally agreed metrics to measure medical education outcomes on social media contributes to the lack of research in the field. At present, publishing on a social media platform does not offer the option of peer review, but as methods of publishing change, traditional publishers will need to embrace this change.

## Conclusions

Innovative methods to inspire, engage, and inform students contributing to the evolution of medical education should be encouraged. Social media platforms are not without their flaws. However, their unique properties allow new innovative teaching-learning experiences to thrive.

## Abbreviations

ASME: Association and Study of Medical Education
